# A multisite cross-sectional study of intercultural competencies in doctor of physical therapy students

**DOI:** 10.1186/s12909-023-04699-y

**Published:** 2023-10-06

**Authors:** Paula A. DiBiasio, Srikant Vallabhajosula, Heidi J. Eigsti

**Affiliations:** 1 Department of Physical Therapy Education, Elon University, Campus Box 2085, Elon, NC 27244 USA; 2 School of Physical Therapy, Regis University, 3333 Regis Blvd, Denver, CO 80221 USA

**Keywords:** Cultural competence, Intercultural competencies, Outcome measure, Global education, Physical therapy

## Abstract

**Background:**

Physical therapists (PTs) work in diverse communities with individuals whose identities and beliefs may differ significantly from their own. Academic institutions must include intentional curriculum aimed at graduating PTs who can skillfully navigate intercultural encounters. Being prepared to engage with difference and demonstrate skills related to intercultural competencies (ICC) will prepare entry-level PTs to provide individualized, high-quality care. Intercultural competencies are essential skills that can reduce healthcare disparities, and promote equitable and inclusive healthcare delivery. This study examined the impact of PT curricula, student demographics, and participation in intercultural learning experiences (ILEs) on students’ development of ICC.

**Methods:**

A cross-sectional study of 8 Doctor of Physical Therapy (DPT) programs in the United States (US) compared ICC in first-year (F) and third-year students (T), and T who participated in an ILE (T + ILE) to those who did not (T-only). Subjects included 1,038 students. Outcome measures included The Inventory for Assessing the Process of Cultural Competence-among healthcare professionals-Student Version^©^ (IAPCC-SV), and a demographic survey.

**Results:**

Independent t-tests showed that group T (mean = 64.34 ± 5.95, 95% CI: 63.78-64.90) had significantly higher IAPCC-SV total scores than group F (mean = 60.8 ± 5.54, 95% CI = 60.33-61.27, *p* < 0.05). Group T + ILE (mean = 65.81 ± 5.71, 95% CI = 64.91-66.71) demonstrated significantly higher IAPCC-SV total scores than group T-only (mean = 63.35 ± 5.8, 95% CI = 62.6-64.1, *p* = 0.039). A one-way ANOVA and post hoc comparisons showed that the 25 to 34-year age group (mean = 63.80 ± 6.04, 95% CI = 63.25-64.35, *p* < 0.001) and the ≥ 35-year age group (mean = 64.21 ± 5.88, 95% CI = 62.20-66.22, *p *< .024) had significantly higher IAPCC-SV total scores, than the 18 to 24-year age group (mean = 60.60 ± 5.41, 95% CI = 60.09-61.11). Students who identified in US census minority ethnic or racial categories (US-Mn) (mean = 63.55 ± 5.78, 95% CI = 62.75-64.35) had significantly higher IAPCC-SV total scores than students who identified in US majority ethnic or racial categories (US-Mj) (mean = 61.98 ± 5.97, 95% CI = 61.55-62.413, *p* = .0001).

**Conclusions:**

Results of the study support the hypothesis that DPT programs can promote the development of intercultural skills in students. The ultimate objective of this academic preparation is to improve the student’s ability to deliver equitable, person-centered healthcare upon entry into practice. Specific ICC for entry-level DPT students are not clearly defined by US physical therapy professional organizations, academic institutions, or accrediting body. Students who participated in an ILE exhibited higher levels of ICC when compared to those who did not. Findings from this study can guide curriculum development, utilization of resources, and outcomes assessment. More research is needed to examine characteristics of an ILE that could inform best practice.

**Supplementary Information:**

The online version contains supplementary material available at 10.1186/s12909-023-04699-y.

## Background

Upon graduation, physical therapists (PTs) may find themselves working in unique, culturally diverse communities with individuals whose identities and beliefs differ significantly from their own. Academic institutions must include intentional curriculum aimed at graduating PTs who can skillfully navigate intercultural encounters in a variety of complex healthcare settings.

Culture has been defined as beliefs, social forms, and traits shared by a group of individuals [1]. Within cultures are diverse concepts of health, illness, wellness, and health-seeking behaviors. These concepts are influenced by sociocultural dimensions such as sexual identity, sexual orientation, ethnic identity, race, language, gender, gender identity, religion, intellectual and physical ability, socioeconomic status, and customs [2]. Understanding the complexity of cultural dimensions of health behaviors and beliefs is essential for the provision of culturally responsive, person-centered care [3]. Person or patient-centered care is described as individualizing care and promoting the individual’s unique needs, views, values, and priorities [4]. Within cultural groups, variation can be significant and homogeneity should not be expected or anticipated. Teaching cultural stereotypes or overgeneralizations, in an effort to bridge intercultural knowledge gaps, is insufficient in preparing graduates to provide culturally responsive, person-centered, and equitable healthcare [5]. “Care cannot be compassionate if culture is not considered, as the interpretation of compassion may vary among and within different cultural groups” [6] p154.

Campinha-Bacote describes cultural competencies as a set of skills essential to patient care that allow healthcare providers to be effective within a patient’s cultural context [7]. Deardorff states that “(I)ntercultural competencies in essence are about improving human interactions across difference whether within a society (differences due to age, gender, religion, socio-economic status, political affiliation, ethnicity, and so on) or across borders” [8] p5. Authors of this study integrated concepts from Deardorff’s definition of intercultural competencies (ICC), with Campinha-Bacote’s healthcare framework [8, 9]. For the purpose of this study, ICC are defined as necessary skills for improving human interactions across difference, that allow a healthcare provider to be effective within a patient’s cultural context, and are essential for promoting the delivery of person-centered, equitable healthcare [8, 9].

Scholars agree that ICC are not an endpoint at which one arrives but develop over time and demonstrate a lifetime commitment to an inclusive worldview [7, 8]. Healthcare providers who have mastery in skills related to ICC adapt their behavior in order to provide individualized, high-quality care to all patients. Intercultural competencies include skills such as; cultural awareness, cultural sensitivity, and cultural humility. Cultural awareness is the foundation of ICC and requires the recognition of individual differences and the potential variables contributing to those differences. Cultural awareness begins with self-reflection and self-critique, and when nurtured, expands from an understanding of one’s self to a consciousness of differences in others [10]. Cultural sensitivity moves beyond cultural awareness and provides a level of respect for differences in all interactions [10]. Cultural humility requires a collaborative dialog between two or more people (e.g. patient-healthcare provider) such that the provider is not solely the receiver of information, but is also cognizant of the power dynamics and impact of their own culture on the encounter [11]. Self-awareness of personal biases is critical for developing this collaborative dialog [6, 12]. Cultural humility allows one to engage in an egoless manner and can result in culturally sensitive and empathetic communication [6, 12]. Cultural awareness, sensitivity and humility do not necessarily occur in a hierarchical order, but interact and contribute to the development of ICC [10, 13].

Intercultural competencies in healthcare providers can reduce healthcare disparities, and promote inclusive healthcare delivery [14]. Effective communication skills have been shown to reduce the risk of cultural misunderstandings that may result in culturally insensitive behaviors and impede equitable healthcare delivery [15]. Providers who demonstrate ICC are able to adapt their behavior, demonstrate empathy, and build trusting patient-provider relationships, which in turn, may reduce disparities in care and promote positive patient outcomes [14]. Graduating PT students who have the confidence to question biases and stereotypes, and the ability to understand and advocate for the unique needs of their patients, have the potential to challenge structures binding inequity, and over time, improve health outcomes. Our research examined students’ development of ICC as an outcome of learning in 8 US Doctor of Physical Therapy (DPT) academic programs.

The American Physical Therapy Association House of Delegates mandates that all members demonstrate cultural competence [16]. The Commission on Accreditation in Physical Therapy Education (CAPTE) criteria include cultural competence as a requirement in DPT program course objectives, learning experiences, and student assessment [17]. The development of ICC must be nurtured via curricula that teaches students to expect, explore and embrace differences in health behaviors and outcomes among people of different cultures. For example, the role of the family in healthcare decision-making and the interpretation of power dynamics in healthcare delivery vary widely among cultural groups and individuals. Programs must prepare students to use a variety of strategies to bridge across differences in the context of culture [18]. Learning experiences should encourage students to explore the role of sociocultural dimensions of health and to practice communication strategies (responsive listening, questioning and clarifying) in settings that integrate self-reflection and formative [5, 14, 16]. To meet objectives related to ICC, physical therapy academic programs are increasingly offering intercultural learning experiences (ILEs), within clinical education, service-learning courses and elective activities. In a survey of DPT academic programs, 77.8% of respondents reported offering an ILE [19]. Some DPT programs offer ILEs in response to students’ desire to study abroad. Many ILEs lack clear goals and student learning objectives [19]. In a study of 56 DPT programs only 55% of the programs with ILEs assessed student learning outcomes and reported using student assignments as the source of assessment [19].

Measuring ICC presents challenges and provides a static snapshot of a lifelong longitudinal developmental process. However, assessment of program goals and learning outcomes is important, and aids in the creation and modification of curriculum, teaching, pedagogy, and learning experiences. Large multisite studies examining the impact of PT curricula and students’ development of ICC have not been published. Within PT programs, students’ ICC have typically been measured in small samples, before and after an ILE. Students who participated in an ILE have never been compared to those who have not, and variables of an ILE have not been examined for their influence on the development of ICC. For the purpose of this study, an ILE was defined as an intentionally designed, culturally unique domestic or international, healthcare-related learning experience in the DPT program, for which participation was voluntary. The ILEs included clinical education, elective courses, and service-learning experiences. Understanding how students develop ICC is vital to enhancing curricular design, assessment of program and student outcomes, and utilization of academic resources. The purposes of this study were to 1) examine the impact of DPT curricula and student demographics (gender identity, age, ethnicity/race) on students’ ICC, 2) investigate the difference in ICC between students who participated in an ILE and those who did not, 3) analyze the contribution of select ILE characteristics (duration, housing, peers, skills practice, learning new skills) to ICC development.

## Methods

A sample of convivence was utilized from faculty in DPT programs who were part of a global health special interest group. Seven DPT programs, in addition to the primary investigator’s institution, agreed to participate. Institutional Review Board approval (protocol #16–132) was obtained at the primary investigator’s institution and at the participating institutions based on their requirements (Regis University, Smithfield College). Seven of the participating institutions were private, all were ground-based programs with a mean class size of 50.75 (SD 22.24). The institutions represented different regions of the US; Northeast (3), Southeast (1), Midwest (2), Northwest (1), and West (1). One faculty member from each DPT program served as a contact point for that institution and assisted with data collection. DPT students provided informed consent prior to participating in the anonymous surveys.

This research utilized a cross-sectional design in which first-year students (F) were compared to third-year students (T), and third-year students who participated in an ILE (T + ILE) were compared to those who did not (T-only).

Students completed the Inventory for Assessing the Process of Cultural Competence-among healthcare professionals-Student Version^©^ (IAPCC-SV) [20] and a demographic survey within 4 weeks of admission (F), and within 4 weeks of graduation (T). A survey was developed by the primary researcher to gather student demographics and characteristics of ILEs that research suggests are related to ICC. The terminology used to describe an ILE was unique to the institution and defined by the represented faculty member so that it was familiar to students in that program (i.e. study abroad clinical, broadening experience, etc.).

Content validity was strengthened by including questions and response options based on published work and vocabulary reflective of diversity, equity and inclusion (DEI) guidelines at the time [21]. Experts in DEI in higher education were consulted for feedback regarding item content, structure and response choices. Surveys were anonymous and did not collect identifying information. The surveys were administered electronically (survey link) or via paper and pencil while students were in a classroom with a faculty member who briefly explained the purpose and importance of the study. Electronic responses were made from a personal device/computer, and all paper copies (completed or not) were placed an envelope which was collected by a student and returned to the faculty member.

The IAPCC-SV is a standardized assessment with established reliability and validity that measures five cultural constructs: cultural awareness, cultural desire, cultural encounters, cultural knowledge, cultural skill and on a 4-point Likert scale [22]. Cronbach's alpha for the overall scale has been reported as 0.783 [22]. Reliability coefficient (alpha) for the five constructs has been reported between 0.67 – 0.74 [22]. Total scores range from 25–80 with higher scores reflecting greater skills in ICC. Scores are translated into four levels (score range) of ICC on a developmental continuum; *cultural incompetence* (20–40), *cultural awareness* (41–59), *cultural competence* (60–74), and *cultural proficiency* (75–80) [20]. Despite the labeling of the continuum, the highest level (*cultural proficiency*) does not indicate completion of learning and development, but rather the accomplishment of student objectives related to ICC [23].

SPSS 24 (IBM Corp., Armonk, NY) was used for statistical analyses. Descriptive statistics mean and standard deviation (SD) were calculated for all variables. Normality was assessed using Shapiro–Wilk test. Student group data for F and T were combined to assess the difference in ICC according to categories of gender identity (choose all that apply: man, non-binary/non-conforming, transgender, woman, prefer to self-describe, do not wish to provide information), age in years (18–24, 25–34, 35–44, 45–54), ethnicity/race (choose all that apply: American Indian or Alaska Native, Asian, Black or African American, Hispanic/Latinx, Native Hawaiian or Pacific Islander, white, other, do not wish to provide information).

A chi-square test for independence was computed to determine whether gender identity, age, and ethnicity/race were independent of group (F vs T) and (T-only vs T + ILE). IAPCC-SV total and construct scores were compared between groups F and T, and T + ILE and T-only. A one-way ANOVA with post hoc analysis was used to compare IAPCC-SV total and construct scores for all students (T and F combined) based on categories of gender identity, age, and ethnicity/race. The T + ILE group IAPCC-SV total and construct scores were compared between subgroups defined by ILE characteristics of housing (lived with host – yes/no), presence of other DPT students (yes/no), duration of ILE (≤ 4 weeks/ ≥ 5 weeks), opportunity to practice PT skills (yes/no), opportunity to learn new PT skills (yes/no). If the data were normally distributed, independent t-tests were conducted for between-group comparisons. If the data were not normally distributed the Mann–Whitney U test was used. A one-way ANOVA, with post hoc analysis was used to assess the difference of the IAPCC-SV total and construct scores for all comparisons with > 2 the independent factors. An alpha value of 0.05 was used to determine statistical significance. Sample size estimation using G-Power software for statistical power of 0.8 was calculated to test for a Type II error [24].

## Results

See Table 1 for demographic information of the 1,038 DPT student participants (*F* = 545, T = 452). Student response rates were 97–100%. Chi-square tests for independence showed that group F had significantly (*p* < .001) younger students and had a greater (*p* = .008) number of students in the US-Mn category compared to group T. There was a significantly (*p* = .002) larger percentage of male students in group T + ILE than in T-only.

**Table 1:** First (F) and Third (T) year student demographics *n* = 1038 (percentiles)

**Category**	**Sub-category**	**F** **(*n* = 545)**	**T** **(*n* = 452)**	**Chi Square *p* value**	**T** **(*n* = 163)**	**T + TLE** **(*n* = 266)**	**Chi Square *p* value**
**Gender Identity**	Male	31.6	31.9	0.28	62.0	79.6	0.002*
	Female	67.3	62.2		37.7	20.4	
	Non-Binary/Non-conforming, or Transgender	0.4	0.0		0.3	0.0	
	Prefer not to say	0.0	0.2		0.0	0.0	
	Unreported	0.7	5.8		0.0	0.0	
**Age**	18–24 years	72.5	12.8	< .001*	14.8	9.7	0.39
	25–34 years	24.2	76.1		79.5	86.0	
	> = 35 years	2.8	5.1		5.7	4.3	
	Unreported	0.6	6.0		0.0	0.0	
**Ethnicity/race**	American Indian or Alaska Native	0.2	0.0				
	Asian	10.3	6.4		7.9	3.2	
	Black or African American	2.8	1.5		2.1	0	
	Hispanic or Latinx	3.9	1.3		1.5	1.1	
	Native Hawaiian or Pacific Islander	0.4	0.2		0.3	0	
	Multiracial	5.7	5.1		5.1	6.5	
	Other	1.5	2.0		2.1	2.2	
	White	75.4	83.4		81	87	
	US Minority (US-Mn)	24.6	16.6	0.008*	19	13	0.208
	US Majority (US-Mj)	75.4	83.4		81	87	

^*^Denote significance (alpha = .05)

Table 2 shows results of the independent t-tests for between-group comparisons of IAPCC-SV scores of first-year vs. third-year students and third-year students who did an did not participate in an ILE. Missing responses to IAPCC-SV items resulted in variable sample sizes.

**Table 2:** T-tests of IAPCC-SV student scores of first year vs third year and third year ILE vs third year only

**IAPCC-SV Constructs**	**First Year (F)**					**Third Year (T)**					
	**N**	**Mean**	**SD**	**95% CI**		**N**	**Mean**	**SD**	**95% CI**		**T-test** ***p***
**Awareness**	537	10.56	0.87	10.49	10.63	445	10.78	0.87	10.70	10.86	.001*
**Desire**	539	13.97	1.71	13.83	14.11	447	14.37	1.56	14.23	14.51	.001*
**Encounters**	539	15.56	1.78	15.41	15.71	445	16.08	1.78	15.91	16.25	.001*
**Knowledge**	542	12.97	2.24	12.78	13.16	445	14.58	2.28	14.37	14.79	.001*
**Skills**	535	7.73	0.41	7.70	7.76	446	8.54	1.48	8.40	8.68	.001*
**Total**	523	60.8	5.54	60.33	61.27	432	64.34	5.95	63.78	64.90	.001*
	**Third Year (T)**										
**IAPCC-SV Constructs**	**T + ILE**					**T-only**					
	**N**	**Mean**	**SD**	**95%CI**		**N**	**Mean**	**SD**	**95%CI**		**T-test *p***
**Awareness**	161	10.93	0.81	10.80	11.06	266	10.66	0.89	10.6	10.8	.002*
**Desire**	163	14.66	1.46	14.44	14.88	266	14.18	1.61	14.0	14.4	.002*
**Encounters**	162	16.31	1.79	16.03	16.59	265	15.91	1.76	15.7	16.1	.039*
**Knowledge**	160	14.99	2.21	14.65	15.33	265	14.27	2.24	14.0	14.5	.001*
**Skills**	161	8.83	1.36	8.62	9.04	266	8.31	1.5	8.1	8.5	.001*
**Total**	155	65.81	5.71	64.91	66.71	261	63.35	5.8	62.6	64.1	.001*

^*^Denotes significance (alpha = .05)

### IAPCC-SV of first-year DPT students (F) compared to third-year students upon graduation (T)

Group T had significantly higher IAPCC-SV total scores, and higher construct scores compared to group F (all *p* < 0.05). Group F percentiles in IAPCC-SV levels were: 1.15% *culturally proficient*, 55.64% *culturally competent*, 43.20% *culturally aware* and none were *culturally incompetent*. Group T percentiles in IAPCC-SV levels were: 5.56% *culturally proficient*, 73.61% *culturally competent*, 20.83% *culturally aware* and none were *culturally incompetent*.

### ILE in DPT program

Thirty-eight percent of third-year students reported participation in an ILE. Students who participated in an ILE demonstrated significantly higher IAPCC-SV total and construct scores compared to those who did not (*p* = 0.039). Most students who reported participating in an ILE did not live with a host family (93%), participated in ILEs with other students (92%), and had an opportunity to practice (90%) or learn (85%) new PT skills as part of the ILE (Table 3). IAPCC-SV total or construct scores were not significantly different between groups categorized by ILE characteristics (housing, other students, duration, and ability to practice or learn new PT skills). Sample size estimations to achieve 80% power were 500, 1559, 551, 109 and 145 for each of these ILE characteristics respectively.

**Table 3:** ILE characteristics reported in percentiles *N* = 155

Lived with Host		Other students		Duration		Practice PT skills		Learn New PT skills	
**Yes**	**No**	**Yes**	**No**	**4 weeks**	** > = 5 weeks**	**Yes**	**No**	**Yes**	**No**
14.84	92.90	91.61	8.39	52.90	54.19	90.32	18.06	84.52	14.84

### Gender identity, age, and ethnicity/race

Table 4 shows the results of a one-way ANOVA and post hoc analysis for comparisons of IAPCC-SV total and construct scores for, gender identity, age and ethnicity/race categories in combined groups F and T. Due to the small number (*F* = 0.04%, T = 0.0) of students who identified as non-binary/non-conforming, or transgender, two gender identity groups (male/female) were included to increase statistical power. Students identifying as male scored significantly higher than students identifying as female on the construct of cultural skills (*p* = 0.025). Results showed that students in the 25 to 34-year age category had significantly higher IAPCC-SV total and construct scores compared to students in the 18 to 24-year age group (all *p* < 0.001). Students in the ≥ 35-year age category demonstrated higher IAPCC-SV total, cultural encounter and cultural desire construct scores (*p* < = 0.024) compared to students in the 18 to 24-year age category.

**Table 4:**
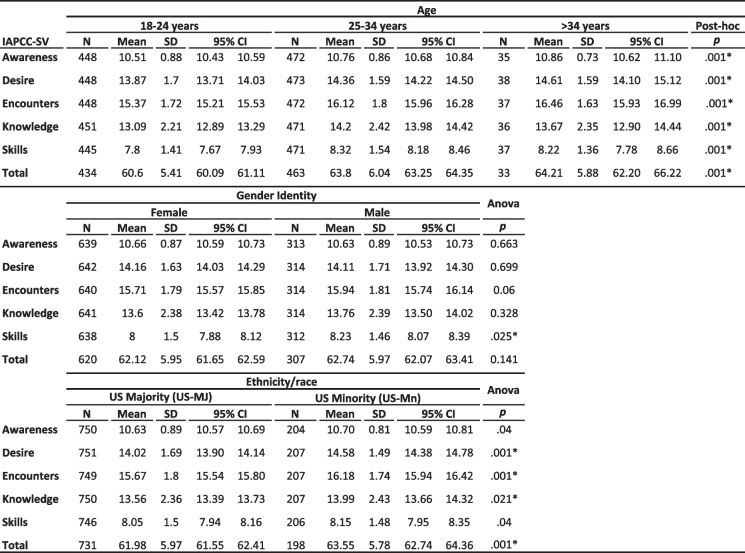
One way Anova & post-hoc analysis of IAPCC-SV scores for all participants categorized by age, gender identity and ethnicity/race

^*^Denotes significance (alpha = .05)

A small number of students identified in individual historically marginalized ethnic/racial categories (0.0 – 10.3%). Therefore, two ethnic/racial categories were created based on US census reports [25, 26], US-Minority (US-Mn) and US-Majority (US-Mj) to increase statistical power [27]. US-Mj consisted of students who identified as white. US-Mn consisted of students who identified as American Indian or Alaska Native, Asian, Black or African American, Hispanic/Latino, Native Hawaiian or Pacific Islander, multi-racial, or other. The students in the US-Mn category had significantly higher IAPCC-SV total (*p* = 0.001) and construct scores for cultural knowledge (*p* = 0.021), encounters (*p* < 0.001) and desire (*p* < 0.001) than students in the US-Mj category.

## Discussion

This study explored the intercultural learning path for DPT students in eight US academic programs. Students in their third-year of study demonstrated significantly higher ICC compared to DPT students in their first-year, and students who reported participation in an ILE exhibited higher levels of ICC when compared to those who did not.

Four previous studies examined intercultural skills in PT students over time [28–31]. One study examined cultural responsiveness in students enrolled in PT Bachelor’s and Master’s programs in Australia and New Zealand [31]. The authors reported no differences in cultural responsiveness at the Master’s level, however, students in their fourth-year of a Bachelor’s program were found to have significantly lower levels of cultural responsiveness than first and second-year students. This difference was attributed to fourth-year students having more experience with diversity, reflection, and self-evaluation resulting in an increased awareness of the skills they were lacking. Doherty et al. [30] found no difference in ICC of first, second and third-year DPT students in one US program. However, the small sample size was a substantial limitation. In another US institution with a 6.5-year (undergraduate entry) DPT program, ICC were assessed in the first, third and sixth-year students [29]. Results showed a positive trend of increasing ICC but without statistical significance between cohorts. This study also had a small sample size, limiting statistical power. Lastly, a longitudinal study following two DPT cohorts, assessed students’ ICC at the beginning and end of the program with the IAPCC-SV [28]. Both cohorts demonstrated statistically significant increases over time, indicating a positive impact of the curricula. Mean IAPCC-SV total scores for the first and third-year students in that study were relatively similar to this study with third-year students’ mean IAPCC-SV total scores in the third level (*cultural competence*) of the IAPCC-SV [20].

Over a third (38%) of DPT students in this study reported participating in an ILE and the majority (85–90%) of these students reported having an opportunity to practice and learn new PT skills during the ILE. Results suggest that efforts and resources required to create and maintain ILEs that enable DPT students to practice and acquire new skills also promote the development of ICC. These results are consistent with published research reporting the positive impact of ILEs on ICC in healthcare students, suggesting a positive return on investment of resources to build high quality ILEs within healthcare academic programs [32, 33]. The Association of American Colleges and Universities has designated ILEs as a High Impact Practice [34] and yet less than 50% of DPT programs in the US provide these opportunities [19]. Embedding ILEs in PT curricula would afford students an opportunity to practice and receive feedback on culturally responsive care in culturally rich and complex environments. Ultimately, students might develop intercultural skills that could contribute to improved health outcomes for underserved and marginalized members of society. These findings are consistent with previous studies examining ILEs that demonstrated significant student development in intercultural skills [35, 36]. Further research is needed with larger sample sizes that investigates which characteristics of ILEs (setting, duration, housing, etc.) are most beneficial to the development of ICCs.

The impact of student demographics on the development of ICC has been studied in healthcare students. Students in this study who identified as male scored higher on the IAPCC-SV constructs of cultural skills and cultural encounters. These findings are incongruent with results of a study of physician assistant students where male students had lower ethnocultural empathy scores but consistent with a study of ICC in nursing students where males had significantly higher ICC [37, 38]. There does not appear to be a theoretical framework to explain gender identity in relationship to ICC in any population. All three studies explored ICC with different measures which may have contributed to the inconsistent conclusions.

There was a significant difference in gender identity between groups T-only and T + ILE with more students identifying as male in the T + ILE group. Given students identifying as male scored significantly higher on the construct of cultural skills in the combined F and T group, it is possible that gender identity accounted for some of the variance in the IAPCC-SV scores in T-only vs T + ILE comparisons. More research is needed to analyze how gender identity impacts ICC in healthcare students.

In this study, older students had significantly higher levels of ICC than younger students. It is expected that students in their third year would be older than those in their first year, and it is unknown how much of the variance in IAPCC-SV scores can be accounted for by age alone. Third-year students also had the benefit of the DPT curricula and 38% of these students participated in an ILE. Similar findings associating age and ICC have been found in undergraduate (nursing) and graduate (physician assistant) healthcare students where older students had higher levels of ICC and more multicultural experiences compared to younger students within the same cohort [37, 38]. More research is needed to determine the impact of age on ICC.

Within the T group, there were a larger number of students in the US-Mj who participated in ILEs. This is a common finding in higher education studies examining student participation in ILEs that report lower participation rates in students who are historically marginalized [39]. Research is needed to identify barriers to participation in ILE’s for students who are historically marginalized. Despite a smaller percentage of students in the US-Mn category and greater ethnic/racial diversity within the US-Mn category, this category demonstrated significantly higher ICC than students in the US-Mj category. Hartman et al.’ [39] reported that “students of color” had higher levels of “openness to diversity” than white students. In a study of physician assistant students, students who identified as members of historically marginalized ethnic/racial groups ranked higher in multicultural experiences, desire for multicultural experiences, and ethnocultural empathy than white students [37]. These results suggest that efforts to recruit and graduate students who are historically marginalized could enhance the PT profession’s ability to meet societal needs [40].

Many professional organizations emphasize the importance of gaining or developing ICC but there are no clearly defined skills or competencies for the PT profession. Given ICC are included in the APTA’s position documents, expectations for the profession, and the CAPTE requirements, educators bear a responsibility to include ICC in the curricula, and to assess ICC in order to assure program initiatives are meeting intended outcomes [41]. However, there is no consensus on best practice for promoting the development of or assessing ICC in PT or other healthcare professional education.

In the past decade, no intervention studies examining the impact of curricula, ILEs, or other learning activities have resulted in student achievement of IAPCC-SV scores in the highest level (*cultural proficiency*) [28, 30, 42–44]. More research is needed to determine the relationship between IAPCC-SV scores and culturally responsive patient-centered care. Because the developmental trajectory of ICC is a lifelong process unique to the individual, healthcare entry-level programs should aim to teach students the reflective practice that is necessary for ongoing professional development. There is a version of the IAPCC for practicing clinicians suggesting higher-level skills are expected with experience. Experts in global education and ICC emphasize that there is no one-size-fits-all approach and recommend creating student learning objectives, targeted interventions, and outcome assessment [41]. It appears important, when assessing healthcare students, to select a measure of ICC that is suited to the healthcare environment. Researchers found only small to negligible correlations when comparing a healthcare specific measure of ICC to a more generalizable measure of ICC within a worldview [45]. These findings suggest that different ICC measures assess different constructs and therefore, it is essential to assess the ICC of PTs using a measure relevant to a healthcare setting. Triangulating data from several sources is highly recommended and considered to more holistically capture students’ intercultural skills [41]. Qualitative data such as student assignments or reflection papers, in combination with quantitative data from standardized assessments of ICC in a healthcare setting *and* in a greater worldview, would be optimal to assess student learning objectives and programmatic outcomes.

## Limitations

Because the IAPCC-SV is a self-report scale, it reflects the student’s self-perception of their ICC and not an instructor’s observation or a patient’s perceived experience. Furthermore, this study utilized a cross-sectional design and did not follow the same students longitudinally, limiting inferences of cause and correlation. Detailed information about each DPT programs’ curricula were not examined as co-variates, therefore, confounding variables, such as a service-learning focus or the presence of a program’s pro bono clinic, may have influenced the data. Specifics of ILEs beyond what was reported, including whether an ILE was faculty led, etc., were not known and therefore may have influenced the results. US DPT programs do not share standardized curriculum therefore much is unknown about the difference between participating programs in this study and other DPT programs in the US. Findings from this research may not be generalizable to all PT programs in and outside the US, especially where terms and concepts of ICC vary significantly as a result of different historical, systemic and structural inequities.

## Conclusions

All DPT programs’ curricula in this study promoted the development of intercultural skills in students. These findings support the objective of advancing the development in ICC throughout a student’s DPT academic experience with the ultimate goal of promoting the provision of person-centered, equitable healthcare as a new graduate. Specific ICC for entry-level DPT students are not clearly defined by physical therapy professional organizations, academic institutions, or accrediting body. Defining ICC as student and programmatic outcomes must be investigated to facilitate the development of curricula that supports student learning. Efforts and resources to create and maintain intercultural learning experiences within DPT programs support the practice and attainment of new PT skills, in addition to the development of ICC. More research is needed to examine specific characteristics of an ILE that inform best practice.

### Supplementary Information

**Additional file 1.** Sample Demographic Survey.

## Data Availability

The data analyzed during this study is not publicly available due to its proprietary nature and the privacy of collaborating institutions. Data are available from the corresponding author upon reasonable request.

## Abbreviations

APTA: American Physical Therapy Association
CAPTE: Commission on Accreditation in Physical Therapy Education
DEI: Diversity, equity and inclusion
DPT: Doctor of physical therapy
F: First-year students
IAPCC-SV: Inventory for Assessing the Process of Cultural Competence-among healthcare professionals-Student Version© (IAPCC-SV)
ICC: Intercultural competencies
ILE: Intercultural learning experience
PT: Physical Therapy
PTs: Physical Therapists
SD: Standard deviation
T: Third-year students
T + ILE: Third-year students who participated in an intercultural learning experience
T-only: Third-year students who did not participate in an intercultural learning experience
US: United States
US-Mj: Unites States ethnic/race Majority based on US census reports
US-Mn: Unites States ethnic/race Minority based on US census reports
