# Supplementary material for: A multisite cross-sectional study of intercultural competencies in doctor of physical therapy students
Source: BMC Med Educ. 2023 Oct 6;23:741. doi: 10.1186/s12909-023-04699-y (PMC10559602; doi:10.1186/s12909-023-04699-y)
Supplement: Supplementary file 1 — Additional file 1. Sample Demographic Survey. [file 12909_2023_4699_MOESM1_ESM.docx]

Sample Demographic Survey

1. What is your current age?

1. Under 18
2. 18 - 24
3. 25 - 34
4. 35 - 44
5. 45 - 54

2. Please indicate your gender identity, select all that apply.

1. Man
2. Non-binary/non-conforming
3. Transgender
4. Woman
5. Prefer to self-describe ____________________________
6. Do not wish to provide this information

3. Please indicate your ethnicity/race.  Check all that apply.

1. American Indian or Alaska Native
2. Asian
3. Black or African American
4. Hispanic/Latinx
5. Native Hawaiian or Pacific Islander
6. White
7. Other __________________________________________________
8. Do not wish to provide this information

4. Within your DPT curriculum did you participate in a Global Learning Opportunities (GLO) international or US course (selective or clinical rotation)? Please indicate all that apply and indicate a number if you participated in either program more than once.

1. US GLO clinical rotation __________
2. GLO selectives __________
3. did not participate in either

Skip To: Q21 If Within your DPT curriculum did you participate in a Global Learning Opportunities (GLO) internati... = did not participate in either

5. Please indicate when your GLO experience/s occurred in your DPT curriculum.

1. 1st year
2. 2nd year
3. 3rd year

6. Did other students from your program participate in the same DPT GLO experience at the same time as you?

1. Yes
2. No

7. Where did you live during your DPT GLO experience(s)?,

1. Dorm
2. Host family
3. Other __________________________________________________

8. What was the duration of your DPT GLO experience(s)?

1. 2 weeks or less
2. 3-4 weeks
3. 5-6 weeks
4. 7-8 weeks
5. 9-10 weeks
6. 11-12 weeks
7. 13-14 weeks
8. 15-16 weeks
9. 17-18 weeks
10. 19 weeks or more please indicate ______________________

9. Were you able to practice your PT skills (examination, evaluation, diagnosis, prognosis, intervention…) as a PT student during this GLO experience(s)?

1. Yes
2. No

10. Did you have the opportunity to learn new PT skills (examination, evaluation, diagnosis, prognosis, intervention…) during this GLO experience(s)?

1. Yes
2. No
